# Bactericidal Efficacy of the Combination of Maresin-like Proresolving Mediators and Carbenicillin Action on Biofilm-Forming Burn Trauma Infection-Related Bacteria

**DOI:** 10.3390/ijms25052792

**Published:** 2024-02-28

**Authors:** Anbu Mozhi Thamizhchelvan, Abdul-Razak Masoud, Shanchun Su, Yan Lu, Hongying Peng, Yuichi Kobayashi, Yu Wang, Nathan K. Archer, Song Hong

**Affiliations:** 1 Neuroscience Center of Excellence, School of Medicine, Louisiana State University Health New Orleans, 2020 Gravier St., New Orleans, LA 70112, USA; 2 Biostatistics, Department of Environmental Health, University of Cincinnati College of Medicine, Cincinnati, OH 45221, USA; 3 Department of Bioengineering, Tokyo Institute of Technology, Box B-52, Nagatsuta-cho 4259, Midori-ku, Yokohama 226-8501, Kanagawa, Japan; 4 Organization for the Strategic Coordination of Research and Intellectual Properties, Meiji University, 1-1-1 Higashimita, Tama-ku, Kawasaki 214-8571, Kanagawa, Japan; 5 Department of Dermatology, Johns Hopkins School of Medicine, Baltimore, MD 21231, USA; ywang107@jhmi.edu (Y.W.); narcher2@jhmi.edu (N.K.A.); 6 Department of Ophthalmology, School of Medicine, Louisiana State University Health New Orleans, 2020 Gravier St., New Orleans, LA 70112, USA

**Keywords:** maresin-like lipid mediator (MarL), specialized proresolving mediators (SPMs), antimicrobial lipids, fatty acids, bactericidal antibacterial, antimicrobial resistance, microbial infections, biofilms, Gram positive, Gram negative

## Abstract

Biofilm-associated bacterial infections are the major reason for treatment failure in many diseases including burn trauma infections. Uncontrolled inflammation induced by bacteria leads to materiality, tissue damage, and chronic diseases. Specialized proresolving mediators (SPMs), including maresin-like lipid mediators (MarLs), are enzymatically biosynthesized from omega-3 essential long-chain polyunsaturated fatty acids, especially docosahexaenoic acid (DHA), by macrophages and other leukocytes. SPMs exhibit strong inflammation-resolving activities, especially inflammation provoked by bacterial infection. In this study, we explored the potential direct inhibitory activities of three MarLs on Gram-positive *(Staphylococcus aureus*) and Gram-negative (*Pseudomonas aeruginosa* and *Escherichia coli*) bacteria in their biofilms that are leading bacteria in burn trauma-related infections. We also examined the effects of MarLs on the bactericidal activities of a typical broad-spectrum antibiotic, carbenicillin (carb), on these bacteria in their preformed biofilms. The results revealed that MarLs combined with carbenicillin can inhibit the survival of Gram-positive and Gram-negative bacteria in their biofilms although MarLs alone did not exhibit bactericidal activity. Thus, our findings suggest that the combination of MarLs and carbenicillin can lower the antibiotic requirements to kill the bacteria in preformed biofilms.

## 1. Introduction

Biofilm-associated infections can lead to major problems in human healthcare industries. Multidrug-resistant bacteria capable of forming biofilms were estimated to be associated with 4.95 million deaths worldwide in 2019 [1]. Biofilms are a cluster of microorganisms embedded in a common niche of extracellular polymeric substance (EPS), and their formation is closely associated with increased resistance to conventional antibiotics and high recalcitrance to immune responses [2,3]. The intricate nature of the biofilms formed by both Gram-positive and Gram-negative bacteria necessitate a thorough investigation into the mechanisms of inherent and acquired resistance to antibiotic treatments and their role in infectious diseases in humans. The leading infective bacteria in burn trauma and other wounds include *Staphylococcus aureus* and *Pseudomonas aeruginosa* [4,5]. Gram-negative *Escherichia coli* is commonly associated with bacterial translocation and sepsis complications due to severe burn injury. Burn wounds are colonized primarily by nosocomial bacterial strains characterized by multidrug resistance and difficulty of eradication, resulting in a major therapeutic challenge [6]. *S. aureus* has been reported as one of the most pervasive Gram-positive microorganisms in burn wounds, accounting for almost 50% of infections [7], while *P. aeruginosa* (17.7%) is the most common culprit in burn wound infections caused by Gram-negative bacteria and *E. coli* is an ubiquitous bacteria critical in the gastro-intestinal systems of severe burn patients [8,9].

Maresin-like specialized proresolving lipid mediators (MarLs) are 14-hydroxyl-containing small molecules transformed enzymatically from essential ω3-docosahexaenoic acid (DHAs) by the cells and tissue of humans or animals [10,11,12,13,14,15]. MarLs include 14*S*,22-dihydroxy-docosa-4*Z*,7*Z*,10*Z*,12*E*,16*Z*,19*Z*-hexaenoic acid (MarL1), 14*R*,22-dihydroxy-docosa-4*Z*,7*Z*,10*Z*,12*E*,16*Z*,19*Z*-hexaenoic acid (MarL2) [13], and 14*S*,21*R*-dihydroxy-docosa-4*Z*,7*Z*,10*Z*,12*E*,16*Z*,19*Z*-hexaenoic acid (MarL3) [10,11,12]. Similar to maresins [13,14,15,16,17,18,19,20,21,22,23,24,25,26,27], MarLs can resolve inflammation and promote tissue regeneration and repair [10,11,12,13]. 

Maresins and MarLs have been extensively studied for their efficacy in resolving inflammation in multiple organ systems, including the cardiovascular [22], digestive [28], immune [29], endocrine [30], nervous [31,32], respiratory [33], reproductive [34], and musculoskeletal systems [35]. Nevertheless, whether their combined use with traditional antibiotics enhances their anti-inflammatory effects remains unknown, especially for biofilm-forming bacteria. Carbenicillin (carb) is a semisynthetic broad-spectrum β-lactam antibiotic that is stable in the presence of gastric acids and potent against a wide range of bacterial strains [36,37]. In treating infections, a nonlethal antibiotic concentration may trigger alternate cellular response pathways leading to increased antibiotic resistance/tolerance [38]. Combining the biofilm-resolving properties of MarL with the bactericidal action of carbenicillin presents a novel innovative therapeutic strategy for addressing the challenges posed by persistent and drug-resistant biofilm-associated infections and improving patient outcomes.

In this study, we investigated the effects of the combination of MarLs and carbenicillin on clinically relevant biofilm-forming *S. aureus* Xen29, *P. aeruginosa* Xen41, and *E. coli* Xen14. *Staphylococcus aureus* Xen29 is a derivative of the parental strain *S. aureus* 12600 that possesses multiple antibiotic resistance [39]. *P. aeruginosa* Xen41 are large microbes (3–5 mm), yellow-green microbes derived from the parental strain *P. aeruginosa* PAO1 [40]. *E. coli* Xen14 are translucent, conical bacterial colonies (~2 mm) derived from the clinical isolate *Escherichia coli* WS2572 parental strain [41], In the face of persistent and drug-resistant biofilm infections, it is critical to investigate the co-actions of maresin-like mediators and carbenicillin as a potential innovative therapeutic strategy for improving the inhibition of bacteria in biofilm that is associated with infections. 

## 2. Results

### 2.1. Determination of Concentration-Dependent Bactericidal Actions of Carbenicillin

**Figure 1:**
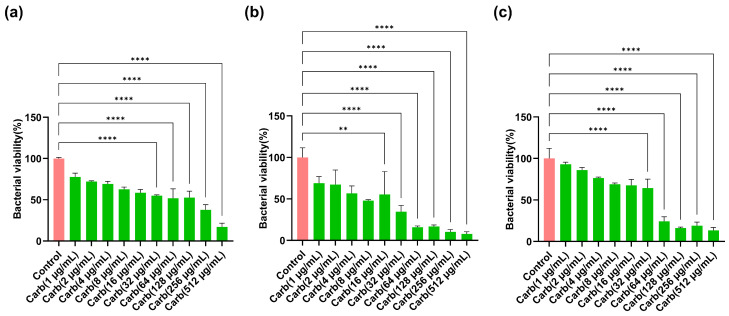
Dose–response graph of carbenicillin inhibition of three key biofilm-forming burn trauma infection-related bacteria: (**a**) *E. coli*, (**b**) *P. aeruginosa*, and (**c**) *S. aureus*. These bacteria were treated in their preformed biofilms. Data are presented as mean ± SEM, *n* = 3. ** *p* < 0.01, **** *p* < 0.0001 vs. control.

Concentration-dependent bactericidal activity of carbenicillin was determined using MTT. *P. aeruginosa*, *S. aureus*, and *E. coli* biofilms formed on 96-well plates. The wells were treated with serial dilutions of carbenicillin to determine a concentration range at which bactericidal effect is recorded. Carbenicillin has been shown to possess increased potency against most Gram-negative bacteria and less so against Gram-positive bacteria; therefore, it is less commonly used for treating Gram-positive bacteria [36,37]. Figure 1 shows that with increasing carbenicillin concentration, the bactericidal activity on preformed bacterial biofilm biomass increased. An approximate *E. coli* cell death of 50% or more was achieved at carbenicillin concentrations of 32 µg/mL or higher. Similarly, 16 µg/mL or higher of carbenicillin treatment resulted in decreased *P. aeruginosa* viability by ≈50%. With respect to *S. aureus*, the carbenicillin concentrations that resulted in approximately 50% bactericidal effect or lower was recorded between 32 µg/mL and 512 µg/mL. Subsequently, for assessing the synergistic properties of MarLs and carbenicillin against bacteria, carbenicillin (32 µg/mL) was chosen for *E. coli* and *S. aureus*, and carbenicillin (16 µg/mL) for *P. aeruginosa*.

### 2.2. Combined Effect of a Maresin-like Mediator and Carbenicillin on Bacterial Viabilities in Their Biofilms

**Figure 2:**
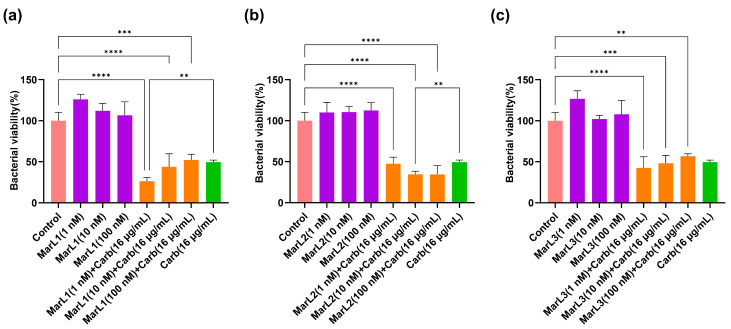
Effects of MarL1, MarL2, or MarL3 at different concentrations on the carbenicillin inhibition of *P. aeruginosa* in preformed biofilms. (**a**) MarL1, MaL2 + Carb 16 µg/mL; (**b**) MarL2, MarL2 + Carb 16 µg/mL; and (**c**) MarL3, MarL3 + Carb 16 µg/mL. Data are presented as mean ± SEM, *n* = 3. ** *p* < 0.01, *** *p* < 0.001, **** *p* < 0.0001.

We used an in vitro preformed biofilm system to examine the potential synergistic effect of MarL1, MarL2, or MarL3 with and without carbenicillin to inhibit bacterial biofilm formation or enhance the carbenicillin’s bactericidal activity. The MTT results showed that, MarL1 at concentrations of 1, 10, or 100 nM combined with 16 µg/mL carbenicillin significantly reduced the optical densities and, thus, the relative amounts of active *P. aeruginosa* compared with control. However, no such effects were seen when compared to carbenicillin (16 µg/mL) treatment alone except for MarL1 in combination with carbenicillin (16 µg/mL), which resulted in a significant reduction in bacterial viability from approximately 50% in carbenicillin (16 µg/mL) group to 25% in the MarL1 (1 nM) + carb (16 µg/mL) group (** *p* < 0.01) as seen in Figure 2a. All three concentrations of MarL2 (1 nM, 10 nM or 100 nM) similarly reduced the amount of viable *P. aeruginosa* significantly compared to the control untreated group (Figure 2b). However, only 10 nM or 100 nM MarL2 + carbenicillin (16 µg/mL) significantly affected bacterial viability in comparison to carbenicillin (16 µg/mL) treatment alone (** *p* < 0.01). With respect to the combination of MarL3 and carbenicillin (16 µg/mL), cell viability was significantly reduced relative to the untreated control group (*** *p* < 0.001, **** *p* < 0.0001), with no such effect seen in comparison with the carbenicillin (16 µg/mL) treatment alone group. Interestingly, the treatment of *P. aeruginosa* with a combined dose of MarL1 (1 nM) + carbenicillin (16 µg/mL) as shown in Figure 2a was as effective as treating the bacteria with a high concentration of carbenicillin (32 µg/mL) as seen in Figure 1b with bacterial viabilities of approximately 26% and 34%, respectively, (*p* = 0.16). Similarly, MarL2 (10 nM or 100 nM) and MarL3 (1 nM or 10 nM) in combination with carbenicillin (16 µg/mL) resulted in a bactericidal effect equivalent to treating *P. aeruginosa* with a high carbenicillin concentration of 32 µg/mL with *p*-values of 0.98 and 0.42, respectively.

**Figure 3:**
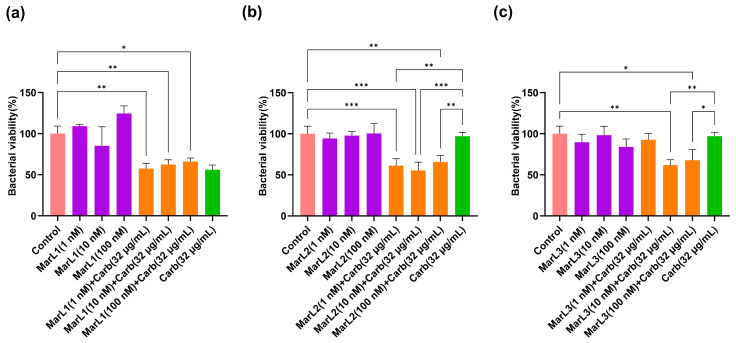
Effects of MarL1, MarL2, or MarL3 at different concentrations on the carbenicillin inhibition of *E. coli* in preformed biofilms. (**a**) MarL1, MarL1 + Carb 32 µg/mL; (**b**) MarL2, MarL2 + Carb 32 µg/mL; and (**c**) MarL3, MarL3 + Carb 32 µg/mL. Data are presented as mean ± SEM, *n* = 3. * *p* < 0.05, ** *p* < 0.01, *** *p* < 0.001.

A similar trend was observed from the results of the MTT test with Gram-negative *E. coli*, where 1, 10, and 100 nM of MarL1 in combination with carbenicillin (32 µg/mL) significantly lowered the relative amounts of metabolically active bacteria present in the biofilms compared to the control group. The viability of *E. coli* reduced from approximately 110%, 105%, and 115% in the MarL1 treatment alone group to 57%, 62%, and 66% in the MarL1 + carbenicillin (32 µg/mL) treatment group at concentrations of 1, 10, and 100 nM, respectively, as seen in Figure 3a. All three concentrations of MarL1 in combination with carbenicillin (32 µg/mL) showed similar efficacy against *E. coli* compared to carbenicillin (32 µg/mL) treatment alone. A combination of MarL2 with carbenicillin (32 µg/mL) demonstrated a relatively improved effect with reductions in bacterial cell viabilities at all three concentrations compared with the untreated control and the carbenicillin (32 µg/mL) treatment alone group as seen in Figure 3b (** *p* < 0.01, *** *p* < 0.001). From Figure 3c, an existing synergy was recorded only in MarL3 (10 nM or 100 nM) combined with carbenicillin (32 µg/mL) as *E. coli* viability reduced in these groups compared to the untreated control group and the carbenicillin (32 µg/mL) treatment alone group (* *p* < 0.05, ** *p* < 0.01). Unlike *P. aeruginosa*, the treatment of *E. coli* with a combined dose of MarL1 (1 nM) + carbenicillin (32 µg/mL) as shown in Figure 3a was as effective as treating the bacteria with high concentrations of carbenicillin (64 µg/mL) and carbenicillin (128 µg/mL) as seen in Figure 1b *p*-values of 0.319 and 0.453, respectively. Additionally, the bactericidal activity of a combined dose of MarL1 (10 nM) + carbenicillin (32 µg/mL) was equivalent to the bactericidal activity of high concentrations of carbenicillin (64 µg/mL) and carbenicillin (128 µg/mL) with *p*-values of 0.216 and 0.150, respectively. MarL1 (100 nM) combined with carbenicillin (32 µg/mL) had a bactericidal efficacy equivalent to treating *E. coli* with carbenicillin (64 µg/mL).

Similarly, MarL2 (1 nM) in combination with carbenicillin (32 µg/mL) was seen to have a bactericidal efficacy equivalent to treating *E. coli* with carbenicillin concentrations as high as 64 µg/mL and 128 µg/mL, with *p*-values of 0.319 and 0.274, respectively. With respect to treatment of *E. coli* with MarL2 (10 nM) +carbenicillin (32 µg/mL), the bactericidal effect recorded corresponded to that of carbenicillin (64 µg/mL, 128 µg/mL, or 256 µg/mL) treatment alone as seen in Figure 1a. The *p*-values recorded were 0.697, 0.724, and 0.059, respectively. 

MarL3 (10 nM) in combination with carbenicillin (32 µg/mL) resulted in a bactericidal effect equivalent to treating *E. coli* with a high carbenicillin concentration of 64 µg/mL or 128 µg/mL with *p*-values of 0.261 and 0.202, respectively. MarL3 (100 nM) combined with carbenicillin (32 µg/mL) was recorded to have a potency against *E. coli* equivalent to carbenicillin (64 µg/mL) (*p* = 0.186) or carbenicillin (128 µg/mL) (*p* = 0.162). 

**Figure 4:**
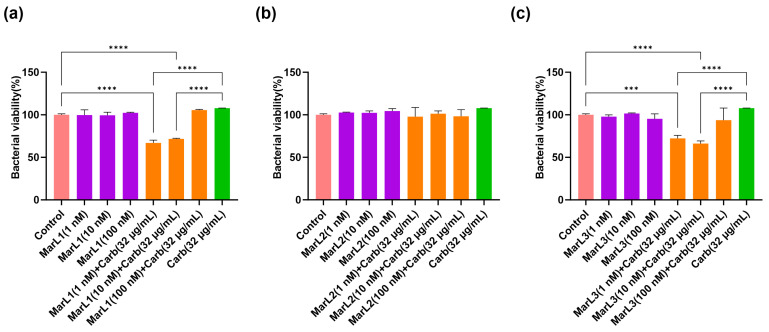
Effects of MarL1, MarL2, or MarL3 at different concentrations on the carbenicillin inhibition of *S. aureus* in preformed biofilms. (**a**) MarL1, MarL1 + Carb 32 µg/mL; (**b**) MarL2, MarL2 + Carb 32 µg/mL; and (**c**) MarL3, MarL3 + Carb 32 µg/mL. Data are presented as mean ± SEM, *n* = 3. *** *p* < 0.001, **** *p* < 0.0001.

From the MTT test results using *S. aureus*, treatment of bacterial biofilm with 1 nM and 10 nM MarL1 combined with carbenicillin (32 µg/mL) significantly reduced bacterial cell viability compared with the untreated control group and with the carbenicillin (32 µg/mL) group with approximately 40% decreases in bacterial cell viability as seen in Figure 4a (**** *p* < 0.0001). On the contrary, MarL2 alone at all three concentrations or in combination with carbenicillin (32 µg/mL) showed no impact on *S. aureus* viability compared with both the control and the carbenicillin (32 µg/mL) treatment alone group as seen in Figure 4b. Again, 1 nM and 10 nM MarL3 combined with carbenicillin (32 µg/mL) significantly reduced the viability of the *S. aureus* biofilm in comparison with the control and the carbenicillin (32 µg/mL) treatment alone group, whereas 100 nM MarL3 combined with carbenicillin (32 µg/mL) showed no such synergistic effect. The observed differences had *p*-values of *** *p* < 0.001, **** *p* < 0.0001.

### 2.3. Live/Dead Assay Imaging Revealed Combined Effects of a Maresin-like Mediator and Carbenicillin on Bacterial Survival in Their Biofilms

A live/dead assay was conducted to validate the antibiofilm activity of the MarLs with or without carbenicillin. Drug penetration and bactericidal activity in the biofilm were assessed through staining with a green SYTO 9 dye, a membrane penetrable dye for both live and dead bacteria with high affinity for DNA, and red propidium iodide dye, which stains nuclear chromatin upon cell membrane disruption (cell death), resulting in fluorescence enhancement.

**Figure 5:**
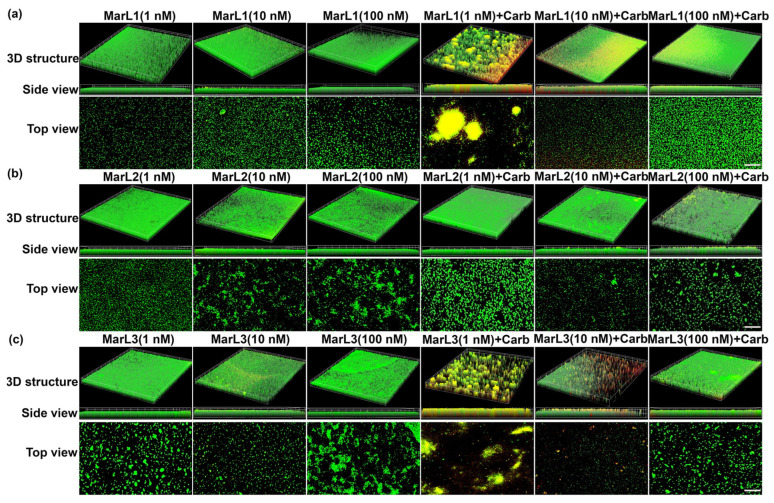
Fluorescence microscopic images showed the effects of MarL1, MarL2, or MarL3 at different concentrations on the carbenicillin inhibition of *S. aureus* in preformed biofilms. (**a**) MarL1 (1, 10, or 100 nM), and MarL1 (1, 10, or 100 nM) + Carb 32 µg/mL; (**b**) MarL2 (1, 10, or 100 nM), and MarL2 (1, 10, or 100 nM) + Carb 32 µg/mL (**c**) MarL3 (1, 10, or 100 nM) and MarL3 (1, 10, or 100 nM) + Carb 32 µg/mL. Green indicates live + dead cells and red indicates dead cells. Images were taken at 4× magnification. Scale bar = 100 µm.

**Figure 6:**
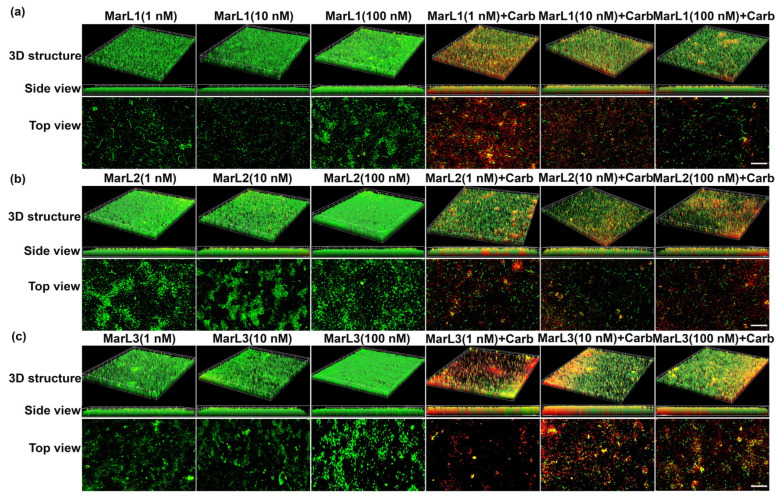
Fluorescence microscopic images showed the effects of MarL1, MarL2, or MarL3 at different concentrations on the carbenicillin inhibition of *P. aeruginosa* in preformed biofilms (**a**) MarL1 (1, 10, or 100 nM), and MarL1 (1, 10, or 100 nM) + Carb 32 µg/mL; (**b**) MarL2 (1, 10, or 100 nM) and MarL2 (1, 10, or 100 nM) + Carb 32 µg/mL (**c**) MarL3 (1, 10, or 100 nM) and MarL3 (1, 10, or 100 nM) + Carb 32 µg/mL. Green indicates live + dead cells and red indicates dead cells. Images were taken at 4× magnification. Scale bar = 100 µm.

**Figure 7:**
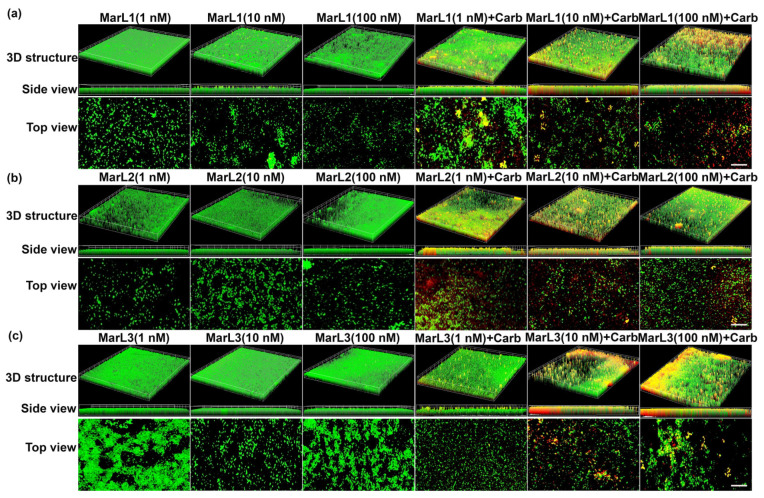
Fluorescence microscopic images showed the effects of MarL1, MarL2, or MarL3 at different concentrations on the carbenicillin inhibition of *E. coli* in preformed biofilms (**a**) MarL1 (1, 10, or 100 nM), and MarL1 (1, 10, or 100 nM) + Carb 32 µg/mL; (**b**) MarL2 (1, 10, or 100 nM) and MarL2 (1, 10, or 100 nM) + Carb 32 µg/mL (**c**) MarL3 (1, 10, or 100 nM) and MarL3 (1, 10, or 100 nM) + Carb 32 µg/mL. Green indicates live + dead cells and red indicates dead cells. Images were taken at 4× magnification. Scale bar = 100 µm.

Treatment with MarL1, MarL2, or MarL3 monotherapy at all three concentrations (1 nM, 10 nM, or 100 nM) exhibited no activity against *S. aureus*, *P. aeruginosa,* and *E. coli* compared with the control group as seen in Figure 5a–c, Figure 6a–c and Figure 7a–c. However, in conjunction with carbenicillin (32 µg/mL), MarL1 (1 nM or 10 nM) positively affected the disruption of *S. aureus* biofilm, with the most effect recorded by MarL1 (1 nM) as seen in Figure 5a. MarL2 in combination with carbenicillin (32 µg/mL) was recorded to disrupt biofilm structure formed by microbially active *S. aureus* at concentrations of 10 nM and 200 nM. MarL3 at all three concentrations of 1, 10, and 100 nM effectively lowered the relative amounts of microbially active bacteria in the biofilms and disrupted the *S. aureus* biofilm most effectively as seen in Figure 1c. 

A similar trend was seen with respect to *P. aeruginosa* and *E. coli*, except that all three MarLs at all three concentrations (1 nM, 10 nM, or 100 nM) were significantly effective at disrupting the formed biofilms with more dead cells visible in Figure 6 and Figure 7 compared to treatments with MarLs only.

In summary, biofilms treated with MarLs alone maintained their integrity and bioactivity, as seen by the uniform green fluorescence with respect to all three bacteria. Concomitantly, an increased number of red-stained dead cells were recorded when bacterial biofilms were treated with corresponding MarLs in conjunction with appropriate concentrations of carbenicillin. This disruption in the biofilm architecture after the combination treatment suggests that MarLs may interfere with the adhesion mechanisms, increasing bacterial susceptibility to carbenicillin. These results were consistent with those of the thiazolyl blue tetrazolium bromide (MTT) assay.

## 3. Discussion

Our study suggests a promising approach for treating biofilm-associated infections via the synergistic actions of MarLs and carbenicillin. Microorganisms, specifically planktonic bacteria, attach to surfaces and multiply based on the characteristics of the substrata and secrete EPS that form biofilms [42]. Biofilm formation is succeeded by the formation of a multilayered defense system comprised persister cells that emerge from dissolved focus areas in the biofilm. This stage is characterized by maximum antibiotic resistance, limited nutrition, subdued antibiotic penetration, and limited proliferation [42,43,44]. Secreted toxins such as lyases and hydrolases also influence the development of antibiotic resistance, as they modify antibiotics into less toxic forms [45,46]. Treatment with antibiotics is effective against planktonic bacteria but not that efficacious against persister cells present in bacterial biofilms. This study provides evidence that Marls have beneficial effects on disrupting biofilms formed by *E. coli*, *P. aeruginosa*, and *S. aureus*. In concert with carbenicillin, MarL1 (1 nM) increased the efficacy of carbenicillin (32 µg/mL) by disrupting biofilm formation and killing *P. aeruginosa* in the preformed biofilm. Higher concentrations of MarL1 (10 nM or 100 nM) in conjunction with carbenicillin (32 µg/mL), though less effective, also disrupted *P. aeruginosa* biofilm formation. MarL2 and MarL3 at all three concentrations were effective at suppressing *P. aeruginosa* biofilm formation with bactericidal activity of at least 50% as seen from the MTT experiment. 

Endogenous SPMs have been documented to possess proresolving properties against bacterial infections [47,48], but the direct mechanism of action of SPMs on microbes is yet to be fully established. Donghoon Kang et al. established the relevance of PqSA, a virulence gene and one of multiple genes [49] implicated in biofilm formation, in the production of cell–cell communication molecules such as 2,4-dihydroxyquinoline [50]. The researchers established that disruption of the PQS biosynthetic protein PqsA affects biofilm formation. Further studies conducted showed that downregulation of PqSA and subsequent disruption of bacterial biofilms was feasible using specialized proresolving mediators, and that solitary treatment with these molecules had no dose-dependent biofilm inhibitory effect [51]. This is consistent with our findings which suggest that the proresolving properties of MarLs disrupted the protective biofilm matrix, increasing antibiotic penetration and rendering the bacteria more susceptible to the bactericidal effects of carbenicillin at low doses. This was evident due to the significant reduction in bacterial cell viability demonstrated by the MTT assays carried out in this study, and further supported by the visual data obtained from the live/dead fluorescence assay. However, further research is warranted to explore the underlying mechanisms. The observed synergy, however, suggests a promising approach for overcoming microbial resistance to antibiotics and for improving the treatment outcomes of biofilm-related infections.

Additionally, further research will target limitations of this study which include extending the research goal beyond the three bacteria used in this research work since our current findings may not be generalizable to all bacteria. Subsequent in vivo studies are also required to accurately represent complexities such as host immune response and other factors that could directly or indirectly affect treatment outcomes. The significance of MarLs in combination with other antibiotics would also be reviewed in our subsequent research.

## 4. Materials and Methods

### 4.1. Materials

*S. aureus* (Xen 29), *P. aeruginosa* (Xen 41), and *E. coli* (Xen 14) strains were generously gifted by PerkinElmer (Waltham, MA, USA). Dimethyl sulfoxide (DMSO), tryptic soy agar (TSA), and carbenicillin sodium salt (CAS No. 4800-94-6) were purchased from Sigma-Aldrich (St. Louis, MO, USA). MTT was purchased from Invitrogen (Catalog number: M6494). Luria-Bertani (LB) broth medium was purchased from Daily Bio (Amherst, NY, USA, product: SD7002). MarL1, MarL2, and MarL3 were prepared through total organic synthesis, and verified and quantified before their usage for the confirmation of their structures, purities, and concentrations using our liquid chromatography coupled with ultraviolet photodiode spectrometry and tandem mass spectrometry as described in our previous publications [10,12,13,14,15]. Prior to use, MarLs were diluted in phosphate-buffered saline (PBS) to the desired concentrations (1, 10, and 100 nM) for the experiments. Carbenicillin sodium salt was prepared as a stock solution from which the following concentrations were made: 512 µg/mL, 256 µg/mL, 128 µg/mL, 64 µg/mL, 32 µg/mL, 16 µg/mL, 8 µg/mL, 4 µg/mL, 2 µg/mL, and 1 µg/mL. LIVE/DEAD BacLight Bacterial Viability Kit was purchased from Thermo Fisher Scientific (Waltham, MA, USA, Cat. No. L7012).

### 4.2. Biofilm Formation

Biofilm was grown on the surface of 96-well microtiter plates according to the following protocols but with revisions [52]. LB broth and TSA were autoclaved for 15 min at 121 °C and brought to room temperature. TSA plates were made by casting 30 mL of the solution into 100 mm petri dishes. From frozen glycerol stocks, bacterial inoculums were made by dipping the loop into the stock solution to scrape bacteria streaking onto corresponding labeled TSA plates. The plates were then incubated overnight at 37 °C. Three individual bacterial colonies were then scooped from each bacterial streaked agar plate into 10 mL liquid LB broth and cultured at 37 °C with mild shaking at 50× *g* overnight. Subsequently, 120 µL of each culture sample were diluted in 6 mL of liquid LB and incubated at 37 °C for 2 h. The optical densities of the microbial suspensions were adjusted to 0.5 McFarland units with turbidity between 0.08–0.1 at 540 nm by adding ice-cold PBS (~1 × 10^9^ colony-forming units [CFU]/mL). This was followed by centrifugation at 3000× *g* for 10 min at 4 °C, carefully decanting the supernatant, and resuspending the pellets in PBS. After a second centrifugation step at 3000× *g* for 10 min at 4 °C, bacterial pellets were resuspended in 1.5 mL of PBS on ice. The bacterial concentration was further verified by serially diluting 500 µL of the bacterial suspensions in cold PBS at concentrations of 1:10, 1:100, 1:1000, 1:10,000, 1:100,000, and 1:1,000,000, and plating 20 µL on an LB agar plate followed by an overnight incubation of the plates at 37 °C in a humidified incubator. CFU were counted by gross examination to calculate the bacterial concentration. Next, 150 µL of bacterial suspensions were added to each well of a 96-well plate and incubated at 37 °C for 24 h without shaking to form biofilms. Fluorescence microscopy was performed on a Discover-Echo Revolve fluorescence microscope (Discover-Echo, San Diego, CA, USA) with an OLYMPUS OLyVIA software version 3.4.1 (Bartlett, TN, USA) was used to assess the formed biofilms.

### 4.3. Determination of Concentration-Dependent Bactericidal Actions of Carbenicillin 

An experiment was conducted to identify the range of carbenicillin concentrations within which a bactericidal effect is achieved. Varying concentrations of carbenicillin (512, 256, 128, 64, 32, 16, 8, 4, 2, and 1 µg/mL) were added to the wells of a 96-well plate containing bacterial biofilms cultured in LB medium as previously described and incubated for 24 h at 37 °C. After washing the wells three times with 1× PBS to remove planktonic bacteria, 10 μL of the 12 mM MTT solution was added to each well. A negative control well was created by adding 10 µL of the 12 mM MTT stock solution to 100 µL of LB medium alone. The plates were incubated for 3 h, followed by adding 150 µL of DMSO, incubating for 10 min at 37 °C, and measuring absorbance values at 540 nm using a SpectraMax M5^®^ spectrophotometer (Molecular Devices, San Jose, CA, USA).

### 4.4. Determination of Bacterial Viability with the MTT Assay

Bacterial cell viability was determined via MTT assay according to Mohamed et al. and Grela et al. with modifications [53,54]. The old medium was removed from the wells containing the biofilms and replaced with MarLs alone or in combination with carbenicillin at concentrations of 1, 10, and 100 nM. Wells containing untreated biofilms served as negative controls, and positive control wells were those treated with carbenicillin (32 µg/mL) only. The plates were incubated at 37 °C for 24 h without shaking. The medium was discarded, and the wells were washed with PBS to remove planktonic bacteria. Next, 10 µL of 12 mM (MTT) were added to each well, with a negative control well included by adding 10 μL of 12 mM MTT to 100 µL of LB medium. After 3 h of incubation, the solutions in the well were replaced with 150 µL of DMSO, incubated for another 10 min at 37 °C, and the absorbance read at 540 nm to quantify formazan—a soluble compound that reflects the relative amount of metabolically active bacterial cells in the biofilm.

### 4.5. Live/Dead Assay 

To assess the integrity of microbial biofilms, microbes were grown on microscope slides by adding 100 µL of bacterial suspensions to each well of an 8-well plate and cultured at 37 °C for 24 h as follows: *P. aeruginosa*—concentration 5.50 × 10^6^ CFU·mL^−1^; *E. coli*—concentration 1.58 × 10^7^ CFU·mL^−1^; and *S. aureus*—concentration 4.8 × 10^6^ CFU·mL^−1^. The wells containing the microbial biofilms were grouped according to the following treatment groups: carbenicillin (32 µg/mL), MarL1, MarL2, and MarL3 at concentrations of 1, 10, and 100 nM, carbenicillin + MarL1 (1 nM, 10 nM, and 100 nM), carbenicillin + MarL2 (1 nM, 10 nM, and 100 nM), and carbenicillin + MarL3 (1 nM, 10 nM, and 100 nM). Untreated wells served as control. After treatment, the plates were incubated at 37 °C for 24 h, followed by washing with 1X PBS and staining using LIVE/DEAD BacLight Bacterial Viability Kit for 15 min at room temperature according to the manufacturer’s protocol. Fluorescence images were obtained on a Discover-Echo Revolve fluorescence microscope (Discover-Echo, San Diego, CA, USA) with Z-stack images used to assess the depth of cell lysis within the bacterial film.

### 4.6. Statistical Analysis

Data were analyzed by one-way ANOVA using Graphpad Prism software (version 10.1.2(324) and R (version 4.3.2) to assess significant differences between groups. *p* < 0.05 was considered statistically significant. Data were mean ± standard error of mean (SEM) of three independent experiments. A *p* < 0.05 was considered statistically significant.

## 5. Conclusions

We investigated the therapeutic activity of MarLs on bacterial biofilms formed by clinically relevant Gram-positive *S aureus* and Gram-negative *P. aeruginosa* and *E. coli*. The metabolic activities in these bacteria inhabiting the biofilms were assessed using the MTT colorimetric method and fluorescence microscopy before and after treatment with MarLs alone and combined with carbenicillin. We identified 16 µg/mL and 32 µg/mL to be the carbenicillin concentration that causes a decrease in the relative amounts of standard *P. aeruginosa*, *S. aureus*, or *E. coli* inoculums, respectively. In other words, the combination helped disrupt biofilm integrity and reduce the relative amounts of microbially active pathogens. Thus, our results revealed that this combination can lower the antibiotic requirements for killing the bacteria in the preformed biofilm. This is especially critical in combating antibiotic resistance since low/nonlethal antibiotic doses are rendered effective by the existing synergy with MarLs.

Our ongoing research delves into the molecular mechanisms underlying this synergy—specifically, the molecular/genetic makeup of bacterial colonies in a biofilm following treatment with MarLs with and without carbenicillin. Future studies should target the broader applicability of combination therapies involving MarLs and antibiotics across varying strains of bacteria, as well as different compositions of bacterial biofilms. Additionally, we plan to explore the role of host-directed MarLs-enhanced carbenicillin effect on immune cell infiltration, anti-inflammation, bacterial clearance, tissue re-epithelization, and healing outcomes in a burn wound infection in animal models.

## Data Availability

Data are contained within the article.

## References

[1] Antimicrobial Resistance C. (2022). Global burden of bacterial antimicrobial resistance in 2019: A systematic analysis. Lancet.

[2] Shineh G., Mobaraki M., Bappy M.J.P., Mills D.K. (2023). Biofilm Formation, and Related Impacts on Healthcare, Food Processing and Packaging, Industrial Manufacturing, Marine Industries, and Sanitation—A Review. Appl. Microbiol..

[3] Costa O.Y.A., Raaijmakers J.M., Kuramae E.E. (2018). Microbial Extracellular Polymeric Substances: Ecological Function and Impact on Soil Aggregation. Front. Microbiol..

[4] Frank D.N., Wysocki A., Specht-Glick D.D., Rooney A., Feldman R.A., St Amand A.L., Pace N.R., Trent J.D. (2009). Microbial diversity in chronic open wounds. Wound Repair Regen..

[5] Tomic-Canic M., Burgess J.L., O’Neill K.E., Strbo N., Pastar I. (2020). Skin Microbiota and its Interplay with Wound Healing. Am. J. Clin. Dermatol..

[6] Hemmati J., Azizi M., Asghari B., Arabestani M.R. (2023). Multidrug-Resistant Pathogens in Burn Wound, Prevention, Diagnosis, and Therapeutic Approaches (Conventional Antimicrobials and Nanoparticles). Can. J. Infect. Dis. Med. Microbiol. = J. Can. Des Mal. Infect. Microbiol. Medicale.

[7] Vinaik R., Barayan D., Shahrokhi S., Jeschke M.G. (2019). Management and prevention of drug resistant infections in burn patients. Expert Rev. Anti-Infect. Ther..

[8] Halstead F.D., Rauf M., Moiemen N.S., Bamford A., Wearn C.M., Fraise A.P., Lund P.A., Oppenheim B.A., Webber M.A. (2015). The Antibacterial Activity of Acetic Acid against Biofilm-Producing Pathogens of Relevance to Burns Patients. PLoS ONE.

[9] Luck M.E., Herrnreiter C.J., Choudhry M.A. (2021). Gut Microbial Changes and their Contribution to Post-Burn Pathology. Shock.

[10] Tian H., Lu Y., Shah S.P., Hong S. (2011). 14S,21R-Dihydroxydocosahexaenoic Acid Remedies Impaired Healing and Mesenchymal Stem Cell Functions in Diabetic Wounds. J. Biol. Chem..

[11] Lu Y., Tian H., Hong S. (2010). Novel 14,21-dihydroxy-docosahexaenoic acids: Structures, formation pathways, and enhancement of wound healing. J. Lipid Res..

[12] Tian H., Lu Y., Shah S.P., Wang Q., Hong S. (2012). 14S,21R-dihydroxy-docosahexaenoic acid treatment enhances mesenchymal stem cell amelioration of renal ischemia/reperfusion injury. Stem Cells Dev..

[13] Hong S., Lu Y., Tian H., Alapure B.V., Wang Q., Bunnell B.A., Laborde J.M. (2014). Maresin-like lipid mediators are produced by leukocytes and platelets and rescue reparative function of diabetes-impaired macrophages. Chem. Biol..

[14] Hong S., Lu Y., Morita M., Saito S., Kobayashi Y., Jun B., Bazan N.G., Xu X., Wang Y. (2019). Stereoselective Synthesis of Maresin-like Lipid Mediators. Synlett.

[15] Nishimura K., Sakaguchi T., Nanba Y., Suganuma Y., Morita M., Hong S., Lu Y., Jun B., Bazan N.G., Arita M. (2018). Stereoselective Total Synthesis of Macrophage-Produced Prohealing 14,21-Dihydroxy Docosahexaenoic Acids. J. Org. Chem..

[16] Serhan C.N., Yang R., Martinod K., Kasuga K., Pillai P.S., Porter T.F., Oh S.F., Spite M. (2009). Maresins: Novel macrophage mediators with potent antiinflammatory and proresolving actions. J. Exp. Med..

[17] Ogawa N., Tojo T., Kobayashi Y. (2014). Synthesis of maresin 1 and (7S)-isomer. Tetrahedron Lett..

[18] Ogawa N., Amano T., Kobayashi Y. (2021). Synthesis of Optically Active Maresin 2 and Maresin 2_n-3 DPA_. Synlett.

[19] Serhan C.N., Sulciner M.L. (2023). Resolution medicine in cancer, infection, pain and inflammation: Are we on track to address the next Pandemic?. Cancer Metastasis Rev..

[20] Serhan C.N., Chiang N. (2023). Resolvins and cysteinyl-containing pro-resolving mediators activate resolution of infectious inflammation and tissue regeneration. Prostaglandins Other Lipid Mediat..

[21] Chiang N., Libreros S., Norris P.C., de la Rosa X., Serhan C.N. (2023). Maresin 1 activates LGR6 receptor promoting phagocyte immunoresolvent functions. J. Clin. Investig..

[22] Serhan C.N., de la Rosa X., Jouvene C. (2019). Novel mediators and mechanisms in the resolution of infectious inflammation: Evidence for vagus regulation. J. Intern. Med..

[23] Serhan C.N. (2017). Treating inflammation and infection in the 21st century: New hints from decoding resolution mediators and mechanisms. FASEB J..

[24] Colas R.A., Dalli J., Chiang N., Vlasakov I., Sanger J.M., Riley I.R., Serhan C.N. (2016). Identification and Actions of the Maresin 1 Metabolome in Infectious Inflammation. J. Immunol..

[25] Li H., Li X., Hao Y., Wu C., Fu Y., Su N., Chen H., Ying B., Wang H., Su L. (2022). Maresin 1 intervention reverses experimental pulmonary arterial hypertension in mice. Br. J. Pharmacol..

[26] Chiang N., Serhan C.N. (2020). Specialized pro-resolving me-diator network: An update on production and ac-tions. Essays Biochem..

[27] Norris P.C., Libreros S., Serhan C.N. (2019). Resolution metabolomes activated by hypoxic en-vironment. Sci. Adv..

[28] Rodriguez M.J., Sabaj M., Tolosa G., Herrera Vielma F., Zuniga M.J., Gonzalez D.R., Zuniga-Hernandez J. (2021). Maresin-1 Prevents Liver Fibrosis by Targeting Nrf2 and NF-κB, Reducing Oxidative Stress and Inflammation. Cells.

[29] Ruiz A., Sarabia C., Torres M., Juarez E. (2019). Resolvin D1 (RvD1) and maresin 1 (Mar1) contribute to human macrophage control of *M. tuberculosis* infection while resolving inflammation. Int. Immunopharmacol..

[30] Sugimoto S., Mena H.A., Sansbury B.E., Kobayashi S., Tsuji T., Wang C.H., Yin X., Huang T.L., Kusuyama J., Kodani S.D. (2022). Brown adipose tissue-derived MaR2 contributes to cold-induced resolution of inflammation. Nat. Metab..

[31] Emre C., Arroyo-Garcia L.E., Do K.V., Jun B., Ohshima M., Alcalde S.G., Cothern M.L., Maioli S., Nilsson P., Hjorth E. (2022). Intranasal delivery of pro-resolving lipid mediators rescues memory and gamma oscillation impairment in App(NL-G-F/NL-G-F) mice. Commun. Biol..

[32] Sanchez-Fernandez A., Zandee S., Mastrogiovanni M., Charabati M., Rubbo H., Prat A., Lopez-Vales R. (2022). Administration of Maresin-1 ameliorates the physiopathology of experimental autoimmune encephalomyelitis. J. Neuroinflamm..

[33] Pan J., Li X., Wang X., Yang L., Chen H., Su N., Wu C., Hao Y., Jin S., Li H. (2021). MCTR1 Intervention Reverses Experimental Lung Fibrosis in Mice. J. Inflamm. Res..

[34] Falsetta M.L., Wood R.W., Linder M.A., Bonham A.D., Honn K.V., Maddipati K.R., Phipps R.P., Haidaris C.G., Foster D.C. (2021). Specialized Pro-Resolving Mediators Reduce Pro-Nociceptive Inflammatory Mediator Production in Models of Localized Provoked Vulvodynia. J. Pain.

[35] Huang R., Vi L., Zong X., Baht G.S. (2020). Maresin 1 resolves aged-associated macrophage inflammation to improve bone regeneration. FASEB J..

[36] Butler K., English A.R., Ray V.A., Timreck A.E. (1970). Carbenicillin: Chemistry and mode of action. J. Infect. Dis..

[37] Castle S.S., Enna S.J., Bylund D.B. (2011). Carbenicillin. xPharm: The Comprehensive Pharmacology Reference.

[38] Bernier S.P., Surette M.G. (2013). Concentration-dependent activity of antibiotics in natural environments. Front. Microbiol..

[39] (2015). Technical Data Sheet, Light Producing Microorganisms—Staphylococcus aureus: S. aureus ATCC 12600 (Xen29).

[40] Caliper LifeSciences (2008). Bioware™ Microorganism—Pseudomonas aeruginosa Xen41-In Vitro Characteristics.

[41] (2023). Technical Data Sheet, Light Producing Microorganisms—Escherichia coli, E. coli WS2572 (Xen14).

[42] Donlan R.M. (2001). Biofilm formation: A clinically relevant microbiological process. Clin. Infect. Dis..

[43] Rather M.A., Gupta K., Mandal M. (2021). Microbial biofilm: Formation, architecture, antibiotic resistance, and control strategies. Braz. J. Microbiol..

[44] Hoiby N., Bjarnsholt T., Givskov M., Molin S., Ciofu O. (2010). Antibiotic resistance of bacterial biofilms. Int. J. Antimicrob. Agents.

[45] Wright G.D. (2005). Bacterial resistance to antibiotics: Enzymatic degradation and modification. Adv. Drug Deliv. Rev..

[46] Schroeder M., Brooks B.D., Brooks A.E. (2017). The Complex Relationship between Virulence and Antibiotic Resistance. Genes.

[47] Chiang N., Dalli J., Colas R.A., Serhan C.N. (2015). Identification of resolvin D2 receptor mediating resolution of infections and organ protection. J. Exp. Med..

[48] Walker J., Dichter E., Lacorte G., Kerner D., Spur B., Rodriguez A., Yin K. (2011). Lipoxin a4 increases survival by decreasing systemic inflammation and bacterial load in sepsis. Shock.

[49] Musken M., Di Fiore S., Dotsch A., Fischer R., Haussler S. (2010). Genetic determinants of *Pseudomonas aeruginosa* biofilm establishment. Microbiology.

[50] Kang D., Turner K.E., Kirienko N.V. (2017). PqsA Promotes Pyoverdine Production via Biofilm Formation. Pathogens.

[51] Thornton J.M., Walker J.M., Sundarasivarao P.Y.K., Spur B.W., Rodriguez A., Yin K. (2021). Lipoxin A4 promotes reduction and antibiotic efficacy against *Pseudomonas aeruginosa* biofilm. Prostaglandins Other Lipid Mediat..

[52] Thibeaux R., Kainiu M., Goarant C. (2020). Biofilm Formation and Quantification Using the 96-Microtiter Plate. Methods Mol. Biol..

[53] Mohamed M.A., Nasr M., Elkhatib W.F., Eltayeb W.N. (2018). In Vitro Evaluation of Antimicrobial Activity and Cytotoxicity of Different Nanobiotics Targeting Multidrug Resistant and Biofilm Forming Staphylococci. Biomed. Res. Int..

[54] Grela E., Kozlowska J., Grabowiecka A. (2018). Current methodology of MTT assay in bacteria—A review. Acta Histochem..

